# Assessing the implementation of a clinical decision support tool in primary care for diabetes prevention: a qualitative interview study using the Consolidated Framework for Implementation Science

**DOI:** 10.1186/s12911-021-01745-x

**Published:** 2022-01-15

**Authors:** Rebekah Pratt, Daniel M. Saman, Clayton Allen, Benjamin Crabtree, Kris Ohnsorg, JoAnn M. Sperl-Hillen, Melissa Harry, Hilary Henzler-Buckingham, Patrick J. O’Connor, Jay Desai

**Affiliations:** 1 Department of Family Medicine and Community Health, University of Minnesota, 717 Delaware Street, Minneapolis, MN 55414 USA; 2 Essentia Institute of Rural Health Research, 502 E 2nd St, Duluth, MN 55805 USA; 3 Department of Family Medicine and Community Health, Rutgers University, 112 Paterson Street, New Brunswick, NJ 08901 USA; 4 HealthPartners Institute, 8170 33rd Avenue South, Bloomington, MN 55425 USA; 5 Present Address: Minnesota Department of Health, 85 East 7th Place, PO Box 64882, St. Paul, MN 55164-0882 USA; 6 Carle Foundation Hospital Clinical Business and Intelligence, 611 W Park Street, Urbana, IL 61801 USA

**Keywords:** Clinical decision support, Consolidated framework for implementation science, Qualitative, Prediabetes, Primary care

## Abstract

**Background:**

In this paper we describe the use of the Consolidated Framework for Implementation Research (CFIR) to study implementation of a web-based, point-of-care, EHR-linked clinical decision support (CDS) tool designed to identify and provide care recommendations for adults with prediabetes (Pre-D CDS).

**Methods:**

As part of a large NIH-funded clinic-randomized trial, we identified a convenience sample of interview participants from 22 primary care clinics in Minnesota, North Dakota, and Wisconsin that were randomly allocated to receive or not receive a web-based EHR-integrated prediabetes CDS intervention. Participants included 11 clinicians, 6 rooming staff, and 7 nurse or clinic managers recruited by study staff to participate in telephone interviews conducted by an expert in qualitative methods. Interviews were recorded and transcribed, and data analysis was conducted using a constructivist version of grounded theory.

**Results:**

Implementing a prediabetes CDS tool into primary care clinics was useful and well received. The intervention was integrated with clinic workflows, supported primary care clinicians in clearly communicating prediabetes risk and management options with patients, and in identifying actionable care opportunities. The main barriers to CDS use were time and competing priorities. Finally, while the implementation process worked well, opportunities remain in engaging the care team more broadly in CDS use.

**Conclusions:**

The use of CDS tools for engaging patients and providers in care improvement opportunities for prediabetes is a promising and potentially effective strategy in primary care settings. A workflow that incorporates the whole care team in the use of such tools may optimize the implementation of CDS tools like these in primary care settings.

*Trial registration* Name of the registry: Clinicaltrial.gov. Trial registration number: NCT02759055. Date of registration: 05/03/2016. URL of trial registry record: https://clinicaltrials.gov/ct2/show/NCT02759055 Prospectively registered.

## Background

Lifetime risk of diabetes in the US now exceeds 40% [1]. Widespread, cost-effective and accessible interventions to prevent or delay diabetes onset a could have a beneficial effect on millions of Americans [1]. Interventions for prediabetes through exercise, diet, weight loss, and targeted pharmacotherapy can prevent or delay progression to diabetes and decrease rates of cardiovascular events [2]. Primary care clinicians (PCCs) are particularly well placed to engage patients in these interventions [3–5]. However, finding ways to mainstream such interventions into routine healthcare delivery is a challenge.

Despite frequent contact between PCCs and patients at risk for diabetes, there are frequent missed opportunities to discuss and address that risk [6, 7]. Less than 23% of patients annually identified with prediabetes were offered any form of referral or treatment to address their condition [6, 8]. There is variability in the attitudes of PCCs on the value of screening for prediabetes, which impacts screening rates [9]. Clinicians may have biases for how motivated and capable their patients might be in relation to undertaking lifestyle changes [9], which also could lead to inconsistent screening practices. However, some studies show that when presented with information about prediabetes and subsequent risk of developing diabetes, patients frequently respond positively and can work to reduce their risk [10]. More research is needed to find more consistent ways to identify and manage prediabetes in primary care practices and real world settings [11], to ensure patients receive information they need about opportunities to reduce their risk.

Clinical decision support (CDS) tools are one strategy for promoting clinical care needs [12] in primary care settings [13], that have been shown to positively impact patient health.(14) Such tools ideally utilize the electronic health record (EHR) at the point of care to identify care gaps and promote evidence-based treatment recommendations [14–16]. However research on the use of CDS tools shows a mixture of success and challenges in making a meaningful and sustained impact on practice and outcomes [12, 17]. Challenges can include a lack of buy in from PCCs as to the value of such tools, particularly if PCCs feel under-informed about the purpose of the CDS [18]. CDS tools that offer advice or recommendations to both PCCs and patients are more likely to be used [12, 19]. CDS alerts to PCCs that can be overridden may be more acceptable [12]. Achieving high use rates is essential to demonstrating improved outcomes due to CDS, but successful integration of the CDS into the workflow is often overlooked and [19], not often achieved in research studies [12]. Research on CDS tools specifically targeting diabetes prevention and cardiovascular (CV) risk factors with prediabetes patients have yet to be published.

Implementation science (IS) studies [20] could offer a valuable perspective to better understand how CDS tools can be optimally incorporated into primary care settings. There is often a gap between how CDS tools are tested in research settings, and how they are successfully implemented and evaluated in practice based settings [21]. The Consolidated Framework for Implementation Research (CFIR) [22] has been used widely in health services research [23] and is ideally suited to evaluate and understand the barriers and facilitators to CDS implementation. CFIR focuses on five key domains in relation to implementation including the intervention itself, the inner and outer settings in which the intervention is implemented, the individuals, and the processes involved in implementation [22].

In this paper we use the CFIR framework [22] to study the barriers and facilitators pragmatic implementation of a web-based, point-of-care, EHR-linked CDS tool designed to identify and provide care recommendations for patients with prediabetes (Pre-D CDS). By utilizing the CFIR framework, the aim of this manuscript is to systematically understand key aspects of CDS implementation in a real world setting that contribute to successful use for the diabetes prevention and reduced CV risk for patients with prediabetes.

## Methods

### Participants and setting

A convenience sample of primary care providers, rooming staff, and clinic managers from 22 Pre-D CDS intervention primary care clinics in Minnesota, North Dakota, and Wisconsin were identified and recruited by the research team to participate in the telephone interviews. The primary care clinics ranged in size from 3 to 25 PCCs based in rural, small town, micropolitan, and metropolitan commuting areas. Eligible participants included PCCs (i.e., family medicine, general internists, physician assistants, and nurse practitioners), clinic and nurse managers, and rooming staff (i.e., RN, LPN, MA). Clinic staff were recruited through e-mails from the health system leadership with follow-up from research study staff and through direct contact by clinic managers. A total of 11 clinicians, 6 rooming staff, and 7 nurse or clinic managers participated in the interviews. The average interview times were 29 min (range 21–42 min), 15 min (range 8–24 min), and 22 min (range 16–32 min), respectively.

### Intervention

Evidence-based algorithms consistent with the American Diabetes Association (ADA) practice standards were used to identify adults at office encounters who met criteria for diabetes screening or laboratory evidence of prediabetes. Algorithms based on the ADA and the American Heart Association/American College of Cardiologists (AHA/ACC) guidelines [24–28] were used to identify individualized care priorities and offer treatment suggestions when appropriate. Cardiovascular risk factors were determined and prioritized using the AHA/ACC pooled 10-year ASCVD risk equation [28]. These CDS suggestions for glucose, lipids, blood pressure, weight, tobacco use, and aspirin use were visually displayed in a low health literacy version for patients and in a high literacy version for PCCs and patients interested in more detailed information. Prior versions of the CDS tool have been tested and published on elsewhere [13].

Workflow implementation of the Pre-D CDS was as follows: (a) during the rooming process triggered by BP entry, EHR information was exchanged with the CDS webservice to identify patients eligible for the Pre-D CDS. For eligible patients, an algorithmically generated best-practice alert (BPA) appeared on the EHR screen within 1 or 2 s; (b) rooming staff clicked on a link within the BPA to display and print the high and low literacy versions of the CDS display; (c) the CDS displays were given to the PCC and the patient to review immediately prior to their visit; and (d) if appropriate, during the visit PCCs and patients reviewed the CDS information and made shared decisions on the care recommendations. The full CONSORT diagram is reported for the intervention elsewhere. This manuscript reports on qualitative data collected from professional implementing the intervention and as such they are not included in the study CONSORT reporting.

### Procedures

A semi-structured interview guide was developed by the research study team (see Table 1). The guide was informed by the CFIR framework, and explored the experience using the CDS in primary care, including its impact on workflow, patient experience, communication during the clinical encounter and implementation of the CDS tool itself.

**Table 1:** Interview guide

1. It would be helpful for me to know about the type of practice setting you are working in, and the type of care you provide. Can you please describe that for me? *Prompt type of clinic, size, type of patients, how long practicing?*
2. Could you walk me through some examples of how you might interact with or use the CDS in a typical day? *Prompt: When do you come across the information, when do you use it, not use it? Why? How well do you feel that works?*
3. Can you please describe to me where the CDS fits in with your current workflow? *Prompt: Did you make any change to make it work, are there changes you would like to see, how might the workflow be better, how does this impact your daily work? Who else in the team do you rely on for it to work?*
4. How have patients responded to the use of the Wizard in the patient encounter? *Prompts: Would they necessarily be aware you had used the Wizard? Use of patient versions? Does the patient seem to be using the patient handout? Have you been sharing the provider version with patients? Why/why not? Which types of patients can you use it with, why/why not? Has the use of the CDS impacted any patient outcomes that you are aware of? Tell me more about that. What comments have pts said? What parts useful, confusing, missing?*
5. Could you tell me about which parts of the Wizard content have you found most useful? Why?
6. Which parts of the Wizard have you found least useful? Why?
Prompts:
* How helpful is the content on treatment recommendations, lab ordering, medication ordering, referrals?*
* How helpful is the content on blood pressure, glucose, aspirin, weight, lipids, smoking?*
* How helpful is the content on screening for breast, lung, colorectal, and cervical cancers? What about getting HPV vaccinations? Do you trust the content to be accurate, up-to-date, and evidence-based? Why or why not?*
* Is there content you would like to see either improved or added to the Wizard? Tell me more about that?*
7. *Do you use the Wizard’s Active Guidelines for ordering? If not, why not? What would make ordering easier?*
8. How has the use of the Wizard influenced your own decision making process during clinical encounters? Can you tell me more about that?
9. How has the use of the Wizard influenced your patients’ conversations with you about cancer and cancer prevention? About prediabetes and cardiovascular disease?
10. I’m curious about how the CDS has impacted you personally in your daily work. Can you describe for me how it has impacted efficiency and/or your experience of your work?
11. Looking ahead to the future, what advice would you share with a new clinic and new providers who are implementing a tool like this? *Prompt: what systems are needed? What training is needed?*
12. How would you like to see the CDS change in the future to support the health of your patients and support you in caring for your patients? *Prompt: Are there problems that need to be fixed? How? What other clinical areas or topics? Lifestyle behaviors? Social determinants of health?*

Semi-structured interviews were conducted by phone or video by members of the research team (DS, CA). The interviews were audio recorded. Verbal consent was obtained immediately prior to the interview, including asking permission to audio record the discussion. Audio recording were transcribed verbatim. No incentives were offered for participation.

### Data analysis

The qualitative data were analyzed using NVivo 12 [29], where one research team member (RP) coded data for emerging themes and sub-themes. The research team used the social constructivist approach to identify themes and sub-themes in the data [30, 31]. This approach allows for themes to emerge from the data, while also considering the broader content of the literature, such as the CFIR framework. Discussions with members of the research team on the emerging analysis further validated the rigor of the qualitative analysis. A consensus approach was used to build agreement about the emerging themes, with any disagreements resolved by discussion and review of the data and coding. The data was initially free coded, without the use of any prior frameworks. During team discussions, the emerging thematic analysis was considered in relation to CFIR and organized by the relevant CFIR domain areas.

### Ethics approval

The Essentia Health Institutional Review Board provided ethical approval for the conduct of this study. Interviewing professionals on their views on the intervention was deemed low risk and therefore a waiver of written consent was granted. An IRB approved information and consent statement was read aloud at the start of each interview, following which verbal consent was obtained from all participants as interview study was considered low risk. The interviewer documented verbal consent in secure study records.

## Results

Here we present the findings of the analysis, with the main themes organized by the CFIR constructs of intervention characteristics, inner setting and process, and their associated sub-themes.

### Intervention characteristics

Four sub-themes of the CFIR construct of the intervention characteristics emerged during the analysis, being evidence strength and quality, relative advantage, complexity and design.

#### Evidence strength and quality

The content of the CDS was generally described as valuable, and reflected information that was consistent with current practice. Some participants expressed neutral opinions, but in general the CDS was positively received. Some participants had specific concerns about the quality of the content, such as feeling that the patient data was not always current. Some felt it would be strengthened by having more input from other interdisciplinary team members, or by having more detailed information. In general, most concerns reflected general controversies in clinical care, such as the best guidelines to use for aspirin recommendations and blood pressure management.

#### Relative advantage

Participants described their views on the relative advantage of using the CDS tool compared with other tools or approaches. Some clinicians were still challenged by the transition from paper-based charts to using electronic tools and technology and struggled with the skills or comfort to use the CDS technology easily.

I think it's a really interesting thing because the transition from paper to computer for me was very challenging, and I put a lot of time and energy into it. I thought I was pretty efficient with the paper chart. I mean, it can't compare to this, of course, but habits die hard. I think as we get more and more stuff-- I'm a little bit overwhelmed. I'm kind of like that guy with a new smartphone, and I need it to do 4 things but it can do 35,000. And it almost takes me half a year to figure out just the 4, to ignore the other 34,996. (P14, PCP)

Some participants felt that they were already awash with tools on their EHR for some of the aspects covered by the CDS, and that there was some concern that PCCs may be at risk of experiencing a certain amount of technology fatigue. There was also concern that the amount of information in the CDS needed to be limited to avoid becoming overly burdensome.

Some clinicians said they were already familiar with using electronic tools that were similar in some aspects to the CDS, including on smartphones. While some continued to express a preference for the tool they were familiar with and had been using, others felt that the CDS offered more convenience by offering one spot where all the information was accessible and pre-populated with patient data. This gave it advantage over other tools, particularly tools that might require a provider to manually enter data.

#### Complexity

Participants indicated that the CDS was fairly easy to integrate into their work, that it was consistently available and had fit into their workflow. Some experienced it as leading to more efficient encounters with patients, particularly when the tool presented useful information in an easily accessible way for both the patient and provider. Participants also described how the CDS helped to focus the encounter by helping to prioritize which health behavior changes might have the greatest impact on the patient’s health.

It has led to a change in priorities, triaging problems if you will, for the patients. So that prior to CV (CDS), inevitably, I would spend 80 to 90 percent of the visit discussing glucose readings, insulin adjustment, those sorts of things. And then with the last three minutes say, "No, oh, and by the way, you really should quit smoking. (P15, Primary Care Provider)

#### Design

Overall, participants described a positive view on the design and look of the CDS. The patient version of the tool was particularly well liked as it was considered to be more visually engaging for patients. The visual aspect was described as being useful in helping patients see what their priorities were in addressing health concerns, and how those concerns related to long term risk.

I think it's a very good tool. Especially I like that the fact that the portion that goes to the patient has pictures on it, that kind of clearly identify what's good and maybe what's not so good or what needs improvement. (P11, Licensed Practice Nurse)

### Inner setting

The CFIR construct of the inner setting was reflected in themes that described complexity, relative priority and resources.

#### Compatibility

Participants described mostly using the CDS in wellness or chronic disease management focused visits. In comparison, for those visits that were for acute reasons, or in walk-in clinic setting, it was generally felt that due to time constraints it was not likely they would use the CDS in those encounters.

Participants consistently described valuing how the CDS supported risk based discussions in the clinical encounter, and helped to prioritize which risk factors to address. Participants described using the CDS to help motivate patients to undertake behaviors that could reduce risk through providing personalized information, such as the potential health impact from losing weight or smoking cessation. Some of these behaviors had been addressed by participants over a long period of time with patients, but by connecting the health behavior to the long term risk for stroke or heart attacks, and potential benefit of risk reduction, some participants felt they could make a more meaningful impact with their patients.

I know there's one lady that had a stroke. And I said, "Well, look at here. Your risk of a cardiovascular event including a stroke, will decrease by 4% if you stop smoking." And her mouth just dropped, "Oh, okay. Well, I need to stop smoking then, because I do not want to have another stroke. (P19, Nurse Practitioner)

Participants described deciding to not share the information with patients who seemed unmotivated, or overwhelmed or struggling to make changes, and in contrast finding the CDS as useful in working with patients who had a new diagnosis, were new to the provider and appeared motivated.

#### Relative priority

Use of the CDS was described as having to fit in with the many different tasks that were already required in the clinical encounter, and it was not always seen as the highest priority activity, particularly in the case of acute visits. Participants described the CDS as one of many change initiatives leading to feelings of being overwhelmed b. Some felt there was a disconnect between system administrators and PCCs, with a sense that administrators were not sensitive enough to the implications of having many new initiatives being rolled out simultaneously. Given the sense of being overwhelmed by many initiatives, some participants stressed the need for more to be done to ensure that PCCs understand the value of the CDS and have a greater buy in into the reasons for using it.

#### Resources

Three key resources were identified as essential to the successful implementation of the CDS. The first of these was training conducted prior to CDS implementation. Virtually no participant could recall the training they had received about the CDS, which may indicate that the tool was particularly straightforward to use technically, and that in an initiative rich environment it may be hard to recall specific training. While it may have been technically straightforward, many participants had failed to retain the reasons why the printed CDS would appear for some patients and not others, and none could recall how to open the CDS manually within the office encounter. Participants shared that further training could have been helpful in the implementation of the CDS, particularly in helping to convey the value and purpose of the CDS.

The second key resource was the availability and location of color printers. The patient version of the CDS used colorful check marks on the screen display, which participants liked. However, most clinics did not have color printers. Additionally, printers were not available in exam rooms and typically the print had to be sent to a printer at a different location within the clinic. This then required the rooming assistant to go to the printer before returning the print out to the appropriate office.

The nurses are supposed to print it off and then put it in with kind of a folder you have when you go into your office. So even though we're paperless, we still have all this paper. Otherwise, if I want to get it, if it hasn't been printed off, I would have to go and print it, request the print. I'd have to leave the room and go and walk down the hall a little ways and pick it off the printer and then come back. So that's very disruptive if it's not there prior to going into the room. (P13, Primary Care Provider)

Finally, time was described a key resource of importance in the implementation of the CDS. Many participants raised concerns that the CDS would take additional time in the consultation, in competition with other priorities, and this may contribute to the CDS mainly being shared in chronic disease management visits.

### Process

#### Engaging

Generally, PCCs described having a positive impression of the CDS and having engaged well with it overall. Participants described the tool as helpful to prioritize discussions in the clinical encounter, and for some, to even motivate them to raise preventative health topics more frequently. It was seen as a useful tool that included relevant information for patient care.

Well, I think it's an interesting-- a group of six variables that has been somewhat helpful to me. My practice tends to be quite geriatric. I deal with a lot of diabetes and cardiovascular disease. I also deal with, of course, a lot of metabolic syndromes and obesity. So, yeah, I would say, in general, it's been an interesting tool that's been of some value. (P14, Primary Care Provider)

The use of the CDS depended heavily on the rooming assistant printing the tool and making it available for the provider, with many describing they usually ensured the tool was ready on the door of the room. Overwhelmingly, the rooming assistants saw their role as printing the CDS for all patients and ensuring it was available for the provider. They did not feel that they were a part of the integration of the CDS beyond that, and they were only helping to facilitate the provider using the tool, even though in contrast, they may be quite active is other wellness visits which were seen to be discussing similar information. There was some variation in practice, with some rooming assistants handing the tool to patients directly and helping to explain the content.

#### Reflecting and evaluating

A range of strategies were described as helping to reflect on engagement and monitor use of the CDS. Participants described utilizing their quality improvement strategies fully in encouraging uptake of the CDS, this included administrator reports on use, print rates, huddles, flow boards and discussions. However, it is noteworthy that the metric of print rates was described as potentially being achieved independent of actual provider use of the CDS. In particular, some clinics found they could always print the CDS, even when the provider has requested they did not, and just shred the tool.

So I work for Dr.X. and he does like to use that as a tool. He does like the print-up. Other PCCs don't use that print-up because they go into the chart and they see it in the chart, is what I was told by my supervisor. Some of them don't like that print-up, so we print it off and then we discard it, shred it. (P25 Medical Assistant)

Finally, when asked about how the CDS might be implemented further or used in different ways in the future, there was enthusiasm for the CDS to be developed for additional clinical topics. This was particularly the case in relation to cancer screening, tobacco and mental health visits. Other suggestions were to better embed the CDS into the patient health record, so patients could either review information ahead of their visit or have the information to access after their visits.

## Discussion

This is the first study to assess the implementation of a CDS tool being used to address diabetes prevention and CV risk reduction in a primary care setting. Diabetes prevention is an important priority [1], and intervening with prediabetic patients is a vital strategy for maximizing diabetes prevention efforts and for early detection and reduction of CV risk [2]. This research has shown that implementing a CDS tool in primary care was feasible and fairly well received. Particularly noteworthy features of the CDS included the ability to support PCCs in talking effectively about risk with patient, and in prioritizing prevention opportunities. The potential scale and reach of such an intervention in primary care could increase opportunities to address diabetes prevention and cardiovascular risk factor management [6, 7].

The CFIR framework [22] was helpful in the systematic identification and description of what was important to PCCs and members of the care team, in implementing the CDS. In this analysis the key domains of intervention characteristics, outer setting, inner setting and process, emerged as relevant to clinic staff. The intervention characteristics were generally considered positively, with the design and content being well liked. There were some concerns that for those who are struggling with the transition to EHR’s from paper-based systems, there could be more support to navigate the technology suggesting consideration of multiple CDS training modes depending on the user’s needs. Alternately, some PCCs used CV risk tools on their smartphones, meaning the CDS had to be more appealing, either through convenience or content, to encourage PCCs away from their phones.

PCCs generally felt patients liked the CDS, but it is noteworthy that PCCs may have preconceived biases about which types of patients might be interested in the CDS information. Patients that were considered unmotivated were seen as less likely to benefit, which could be a challenge for broader CDS integration in patient care. Although, this could also be a potential asset as repeated exposure to the CDS may allow engagement of patients as they become more activated and ready to address diabetes prevention and CV risk factors [32].

The inner setting themes offered an interesting insight into the Pre-D CDS use and integration into current workflows. There was some contradiction between PCCs and members of the care team in relation to how well the CDS was integrated into the workflow. While the PCCs felt it was well integrated, the rooming staff noted how it overlapped with some of their other tasks, particularly in relation to annual wellness visits. This signals an opportunity to ensure such tools are not just provider-centric but engage all members of the care team in its meaningful use. Additionally, there were concerns that the topics covered in diabetes prevention and CV risk management might not always be seen as a patient or provider priority, particularly in the context of acute visits. This raises the question of the timing of CDS tools, and if there is a benefit to tailoring such tools by visit types as well as by patient characteristics.

Finally, the CFIR domain of process offered useful insights into the role of engaging PCCs in the use of the CDS. The findings indicate that while the CDS was technically straightforward, the purpose and value of the CDS may not have been well conveyed during the training and implementation process which could have reduced CDS use. This issue of engagement is reflected in the broader literature [18], and our findings affirm that engagement is an important, and challenging issue for CDS use.

While this is the first study to report on clinic staff perspectives regarding implementation of a diabetes prevention CDS system, there have been several reports on CDS use to improve management of cardiovascular risk factors in primary care patients, a key component of our CDS system [17, 33–35]. The general consensus is that CDS systems addressing cardiovascular risk factors are promising yet evidence of clear outcome benefit and cost-effectiveness remain to be determined. This is likely because the development and implementation of such CDS systems is relatively new, especially given the complexity of delivering patient-centered care [34]. For example, several risk factors need to be managed simultaneously and over time which is a challenge for completeness of risk factor assessment and determining the appropriate pharmacologic and non-pharmacologic treatments, including assessment of contraindications and safety. Furthermore, there is high variability in provider adherence for care guidelines, how care teams function in delivering appropriate and timely care, and how patients are engaged in their care. Potential characteristics of successful CVR CDS systems include effective and automated integration into the clinic workflow, real-time point-of care delivery, provider awareness of the CDS computations and sources of the information, a comprehensive single display the information, provision of specific care recommendations, and engaging patients in the CDS process [33, 34].

The current Pre-D CDS contains most of these successful CDS characteristics and this is echoed in the overall clinic staff satisfaction with its design and content. However, these interviews also highlight the variability in end-user perceptions and use suggesting the need for increased training and communication to reinforce the purpose and value of the CDS and its recommended use in the context of a clinic visit. Increased understanding of the CDS across the clinic team may increase its use and effectiveness.

### Limitations

This study has several limitations. The team that researched the use of the CDS were also involved in the development of the CDS. We have tried to overcome this limitation through the involvement of multiple team members in data collection and analysis, including team members external to the lead institution. Additionally, this may not be a fully representative sample of participants or may reflect concerns that are unique to the health system and setting in which the CDS was being implemented. This may limit the generalizability of these findings.

### Conclusions

The use of CDS tools for providing preventive care for patients with prediabetes offers an important and potentially effective strategy in primary care settings. Focusing on whole care team engagement in the value and use of such tools may optimize the implementation of CDS tools in primary care settings.

## Data Availability

The datasets generated and analyzed during the current study are not publicly available but are available from the corresponding author on reasonable request.

## Abbreviations

ACC: American College of Cardiologists
ADA: American Diabetes Association
AHA: American Heart Association
CDS: Clinical decision support
CFIR: Consolidated framework for implementation research
CV: Cardiovascular
HER: Electronic health record
LPN: Licensed practical nurse
MA: Medical assistant
PCC: Primary care clinicians
PCP: Primary care provider
Pre-D CDS: Prediabetes clinical decision support
RN: Registered nurse
